# Supplementary figures and images for: Improving old tricks as new: Young adults learn from repeating everyday activities
Source: PLoS One. 2023 May 11;18(5):e0285469. doi: 10.1371/journal.pone.0285469 (PMC10174589; doi:10.1371/journal.pone.0285469)

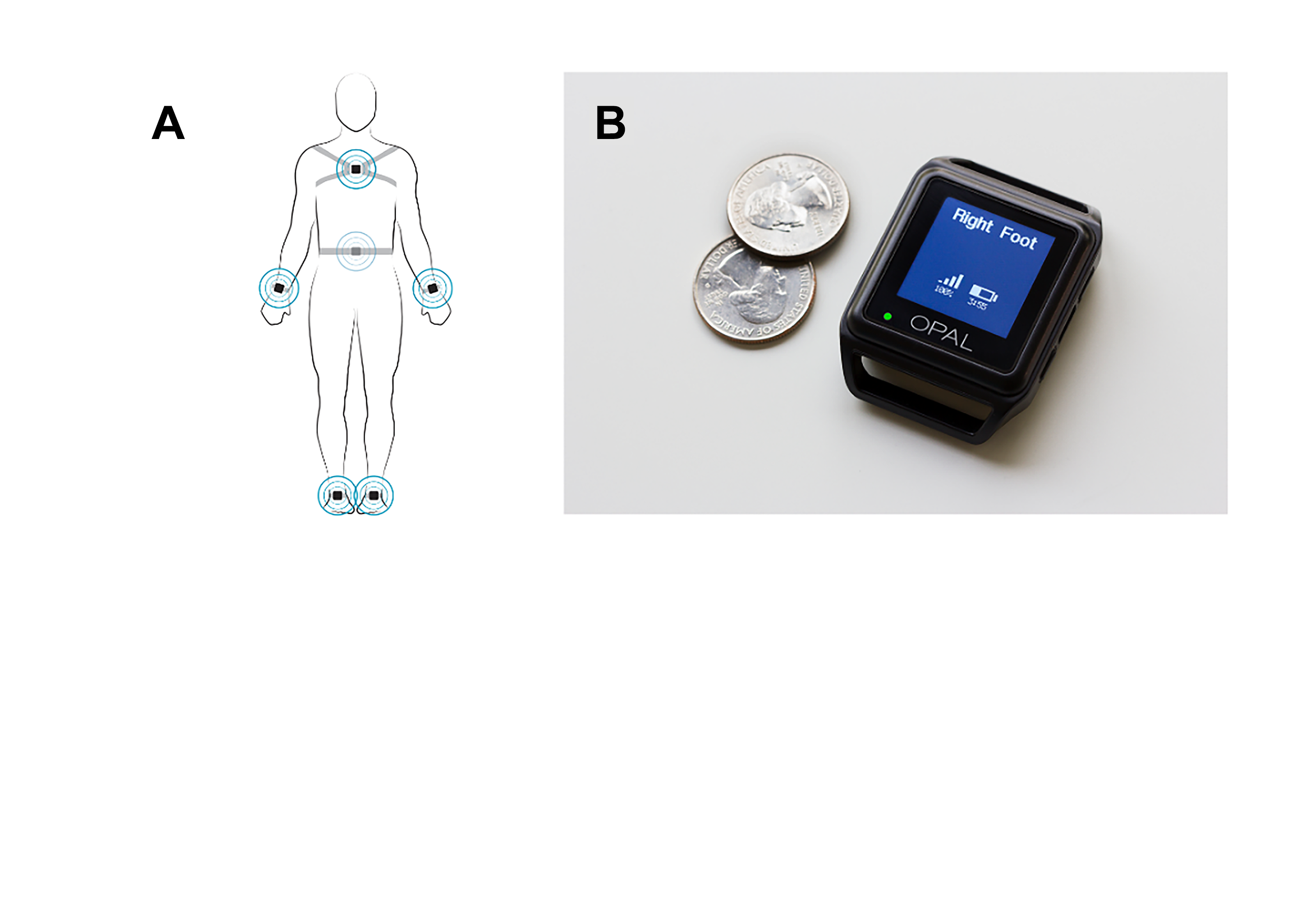

Supplement: S1 Fig — A) Placement of the APDM IMUs, the lumbar IMU (attached to the lower back) is shown in fainter color; B) An APDM IMU. (https://apdm.com/). (TIF) [file pone.0285469.s001.tif]

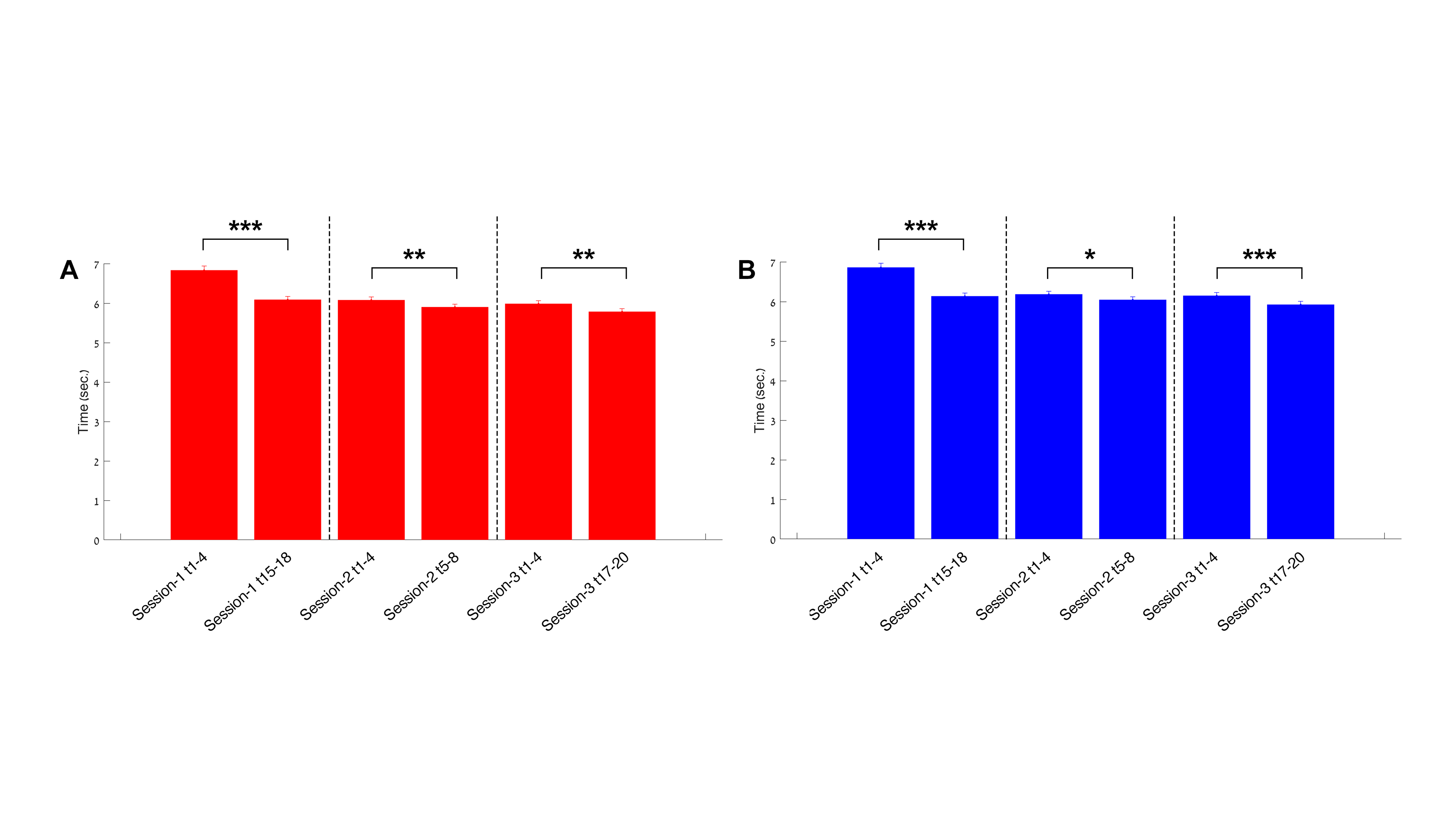

Supplement: S2 Fig — A) According to the videos; B) according to the IMUs. */**/***- p<0.05, p<0.01, p<0.001 (repeated measures ANOVA’s). (TIF) [file pone.0285469.s002.tif]
