# Supplementary material for: Improving old tricks as new: Young adults learn from repeating everyday activities
Source: PLoS One. 2023 May 11;18(5):e0285469. doi: 10.1371/journal.pone.0285469 (PMC10174589; doi:10.1371/journal.pone.0285469)
Supplement: S1 File — (DOCX) [file pone.0285469.s003.docx]

***Practice effects were retained across sessions (video)***

Good retention of the practice effects, across the three sessions, was also apparent when TUG durations were extracted from the video recordings. A comparison of TUG duration, according to the videos, in the final four trials of session-1 to TUG duration in the initial 4 trials of session-2 showed no significant session (F_(1,11)_=0.22, p=0.648, ŋ_p_^2^=0.02). Performance in the two segments was at plateau (F_(3,33)_=1.782, p=0.17, ŋ_p_^2^=0.139). The offline, between sessions, differences in TUG duration were -1.3 ± 4.8, percent for the interval between session-1 and session-2. However, additional significant gains emerged within the second session, i.e., between trials 1-4 and trials 5-8 of the session (F_(1,13)_=11.92, p=0.004, ŋ_p_^2^=0.478). The gains in TUG duration attained by the end of session-2 were well-retained by session-3, i.e., over a one-week interval (F_(1,13)_=0.708, p=0.415, ŋ_p_^2^=0.052). The offline differences in TUG duration from the end of session-2 to the beginning of session-3 were -1.6 ± 4.4, percent. No additional gains in TUG duration accrued between the first two segments of session-3 ((F_(1,14)_=2.162, p=0.164, ŋ_p_^2^=0.134), however, additional gains in TUG duration were accrued after the transfer test segment in session-3, with faster performance in trials 17-20 compared to trials 1-4 of the session (F_(1,14)_=13.85, p=0.002, ŋ_p_^2^=0.497). Overall, the average improvement in TUG duration across the three sessions was 19% (range: 3.3%-28.5%); the average improvement attained before the transfer segment of session-3 was 16.54% (range: 5%-24%).
