# Supplementary material for: Improving old tricks as new: Young adults learn from repeating everyday activities
Source: PLoS One. 2023 May 11;18(5):e0285469. doi: 10.1371/journal.pone.0285469 (PMC10174589; doi:10.1371/journal.pone.0285469)
Supplement: Attachment — Submitted filename: Response to reviewers 6.4.23.docx [file pone.0285469.s018.docx]

April 6. 2023

To: Dr. Peter Andreas Federolf   
Academic Editor, *PLOS ONE*

Ref: PONE-D-22-15151R1

Title: Improving old tricks as new: young adults learn from repeating everyday activities

Dear Dr. Federolf,

Enclosed please find our revised manuscript: "Improving old tricks as new: young adults learn from repeating everyday activities", an original research paper, that has been reviewed as PONE-D-22-15151(R1).

We thank you and the reviewers, again, for the thorough reading of the revised manuscript and for the few further comments. We are pleased to note that our 'large efforts' in revising our 'interesting and important article' is appreciated. We were happy to read that reviewer #1 writes: 'I would like to thank the authors for this interesting study. The authors made large efforts in revising their manuscript. Most of my comments were adequately addressed in the revised version of the manuscript and I think the manuscript substantially improved. Congratulations on this great work', and that reviewer #2 summarized: 'the authors comprehensively addressed all my concerns and I am happy to endorse the publication'.

We have carefully considered the few ‘minor comments’ made by Reviewer 1 and have used these comments to effect some further revisions; we hope you will find the paper suitable for publication in PLOS ONE in its current form.

As requested, we reviewed the reference list and ensured that it is complete and correct. None of the papers cited in the manuscript were retracted.

In the following we address the comments and suggestions made by Reviewer 1 point-by-point. Reviewer 2 had no further comments.

Sincerely,

Gil Leizerowitz, Ran Gabai, Meir Plotnik, Ofer Keren, Avi Karni

**Reviewer #1:**

- “Once again, I would like to thank the authors for this interesting study. The authors made large efforts in revising their manuscript. Most of my comments were adequately addressed in the revised version of the manuscript and I think the manuscript substantially improved. Congratulations on this great work. I have only some minor comments, which the authors may want to address.”

**Response -** We thank the reviewer for this comment. We have carefully considered the reviewer’s remaining minor comments and suggestions and hope that the revised manuscript now addresses these points in the revised manuscript. In the following we explain how we addressed the points raised in your specific comments in a point-by-point manner.

Method:
• Please include a statement on how participants were recruited.

**Response –** We added the following statement to the methods: "The participants were recruited via social media; most participants were university students or staff members at the Sheba Medical Center."

• l. 662-664: Statistical methods are not mentioned for the within-session analysis. Please add.

**Response –** The tests were repeated measures ANOVAs. The text now reads: "Within-session and between-sessions improvements, in terms of TUG duration and kinematics, were assessed by comparing four-trial long segments from the beginning and end of each session. To this end, repeated measures ANOVAs were used with session/segments (time-points) and the trials comprising them as within-subject factors. Within-session gains were assessed by comparing the four-trial long segments from the beginning and end of each session. Between-sessions changes (both overnight and after one week) were assessed by comparing the four-trial long segments from the end of one session and the beginning of the following session."

Results/Discussion:
• Participant awareness of study aims: The authors mentioned that the participants were aware of the aim of the study. Participants may behave differently when they know the research hypothesis. Could it be possible that the participants behaved/performed differently because they knew that the general aim of the study was to “test whether new learning occurs in everyday tasks”. If a participant assumes that learning could occur this might affect performance. I would appreciate if the authors could elaborate on this and at least mention this in the discussion/limitations of the paper.

**Response -** Participants were aware to the aim of the study. They were asked to sign a consent form in order to participate in this study which included the following:

"The purpose of the study is to examine the acquisition and learning of a daily motor skill: getting up from sitting, walking around and returning to sitting position (using a test from the rehabilitation world)."

"The General rational and the aims of the study: Repeated practice can lead to learning and improvement of performance. In the present research we will examine whether and how repeating simple everyday activities affects performance. Performance will be assessed by following movement speed and the characteristics of the movement."

Throughout the three sessions participants were given the same instructions: to execute the TUG test as fast as they can. They were not asked in each trial to achieve better results than in the previous trial. Participants were not made aware of the fact that we are looking into whether gains in performance follow in different phases. Nor were they aware of which of the kinematic measures will be examined. We cannot assume that knowing the aim of the study was central to modifying the participants’ behavior in a specific manner, as found.

In teaching new laboratory motor skill tasks, the participants know the aim of the study and in all tasks what needs to be executed is explicitly stipulated. In most cases participants are also requested to perform as fast and as accurately as possible (Karni et al., 1995). Yet, despite the explicit instructions given to the participants, there is an implicit process of learning of a skill.

Given this comment, and further comments (l. 523-526, l. 527-529) made by the reviewer, we added a short statement: "One should note that participants were made aware of the general aim of study when enrolling in the study, and that the task elements and sequence were explicitly stipulated at the beginning of each session. However, throughout the three sessions participants were given the same instructions - to execute the TUG test as fast as they can without running; participants were not asked to achieve better results than in a previous trial. The participants were not aware that we were looking into whether gains in performance would follow in different phases; nor were they aware of which of the task elements or kinematic measures will be examined. The question of whether knowing the aim of the study was central to modifying the participants’ behavior or that similar learning would occur in a more implicit or incidental learning situation is open for further experimentation. Future studies can also address a possible effect of affording feedback to the participants (e.g., Kilduski & Rice, 2003; Wulf, Shea, & Lewthwaite, 2010), the interaction with the teacher-instructor (e.g., Awad-Igbaria, Maaravi-Hesseg, Admon, & Karni, 2022) or the effects of the length of breaks afforded between the trials (e.g.,Bönstrup, Iturrate, Hebart, Censor, & Cohen, 2020)."

• l. 523-526: The authors mentioned that the participants did not receive feedback on their task performance (i.e., knowledge of result). However, it has been shown that knowledge of result can be fundamental for motor learning. I would appreciate if the authors could elaborate on this and possibly add this to their discussion.

**Response –** We agree. Feedback was found to enhance the learning of a motor skill (Wulf & Lewthwaite, 2016; Wulf et al., 2010) By withholding feedback about the subjects' actual trial-by-trial performance we may have underestimated the potential for task relevant performance gains.

In the new paragraph about possible caveats we enumerate this point: "Future studies can also address a possible effect of affording feedback to the participants (e.g., Kilduski & Rice, 2003; Wulf, Shea, & Lewthwaite, 2010), the interaction with the teacher-instructor (e.g., Awad-Igbaria, Maaravi-Hesseg, Admon, & Karni, 2022) or the effects of the length of breaks afforded between the trials (e.g.,Bönstrup, Iturrate, Hebart, Censor, & Cohen, 2020)."

• l. 527-529 – breaks between trials: It seems that the break duration considerably differed between participants (15 sec. – 19 min). Research has shown that break duration can greatly impact motor learning. Recent studies even suggest that micro-offline learning may occur during short breaks (Bönstrup et al., 2019, 2020). Even though the authors did not compare different groups the variance/heterogeneity in break duration may have influenced the results of the study. Thus, I would appreciate if the authors clearly highlight this in their paper (e.g. limitations). Did the authors analyze if participants’ performance/learning gains systematically differed as a function of break duration? This might be an interesting finding.

**Response –** We report that "The median time between the beginning of one trial and the beginning of the next one was 23 sec. (15 sec. – 19 min, an instance of a IMU problem). The mean break duration was 32 ± 53 sec. (SD)". Both median and average of all the trials breaks were on the order of a half of a minute despite the fact that one break lasted 19 minutes due to technical problems. The difference in break duration was not between participants but rather sparse random occurrences. Only 38 out of 646 breaks [~5%] were longer than one minute. Due to the small number of longer breaks, we cannot perform an analysis on whether participants’ performance/learning gains were affected as a function of break duration. Moreover, the 19 min break did not occur in session-1, it occurred in session-3, well after performance leveled off. However, taking into account the reviewer suggestion, we examined the correlation between the length of session-1 in seconds and 1) the relative improvement in performance in session-1 and 2) the overnight relative improvement in performance. No significant correlation was found (according to either the IMU's or the videos).

We acknowledge the possibility that the length of the breaks between the trials can affect the learning procedure. Recent papers (Bönstrup et al., 2020, 2019) suggested that in early skill learning, short period of rest (on the order of seconds) may result in consolidation. In a follow up study, yet to be published, the participants performed only five iterations of the TUG in the first two sessions; i.e., did not reach the plateau phase within the first session. However, despite the 24 hours break between the first and second session, the learning process continued in the same manner as it had in the presented study. A break of 24hours was not different in terms of TUG duration gains from a ~half a minute long break.

Given the reviewer’s comment, we rephrased the methods section and it now reads:" After completing each trial, data were transferred from IMU-level storage and the participants were asked, and indicated, when they were ready for the next trial. The between-trial breaks were on the order of a half a minute (32 ± 29 sec., mean, SD) with one exception - a 19 min break due to technical problem with the IMU."

Furthermore, we added the possibility to the new caveats-potential future studies paragraph: "Future studies can also address a possible effect of affording feedback to the participants (e.g., Kilduski & Rice, 2003; Wulf, Shea, & Lewthwaite, 2010), the interaction with the teacher-instructor (e.g., Awad-Igbaria, Maaravi-Hesseg, Admon, & Karni, 2022) or the effects of the length of breaks afforded between the trials (e.g.,Bönstrup, Iturrate, Hebart, Censor, & Cohen, 2020)."

• l. 586-591 – additional trials in some participants: Did the authors check if the data of the participant performing more trials differed from the group average. Even though an additional trial during the plateau phase does not influence performance at the end of practice it could influence consolidation (i.e. offline learning).

**Response -** Only two participants were given additional trials: "One participant executed two additional trials (one on session-1 and one on session-2) as replacement trials and another participant executed one replacement trial on session-3". The participants’ gains across the 3 sessions were within 1.2 and 1 SD of the group mean gains.

• l. 191 & 223: please rephrase statistics.

**Response -** The statistics were rephrased in the following manner:

L. 191: “As can be seen in Fig 2 the video and IMUs derived measurements of TUG duration were highly correlated across the three sessions (r_p_=0.858, p<0.001, N=678) and there was no significant difference between the two measures across the 18 trials of the session (F_(1,12)_=0.275, p=0.61, ŋ_p_^2^=0.022).”

L. 223: "A repeated measures ANOVA comparing trials 1-9 to trials 10-18, showed a significant difference between the two phases (F_(1,11)_=69.04, p<0.001, ŋ_p_^2^=0.863; F_(1,11)_=32.519, p<0.001, ŋ_p_^2^=0.747, video and IMU based TUG duration, respectively), a significant Trial effect (F_(8,88)_=12.06, p<0.001, ŋ_p_^2^=0.52; F_(8,88)_=8.77, p<0.001, ŋ_p_^2^=0.424) as well as a significant Phase X Trial interaction (F_(8,88)_=11.24, p<0.001, ŋ_p_^2^=0.506; F_(8,88)_=11.23,p <0.001, ŋ_p_^2^=0.505).”

• I would appreciate if the authors report effect size (e.g. ŋp2, Cohen's d) also for non-significant results.

**Response** – There are ongoing debates about what can be inferred from effect size measures when the effect is not statistically significant (e.g., <https://www.researchgate.net/post/Effect-sizes-for-non-significant-results>). Given the reviewer’s request we added the effect size also for non-significant results.

• l. 259: Please remove quotation mark.

**Response –** The quotation mark was removed.

• l. 265: Please remove the second semicolon.

**Response -** We have removed the second semicolon from the sentence.

• l. 265-269: It is not clear which trials from which session were analyzed here. Please rephrase.

**Response** – We thank the reviewer for the opportunity to clarity of the paragraph. The text now read: "Moreover, additional gains in TUG duration were accrued across session-3, with faster performance in trials 5-8 compared to trials 1-4 of the session (F_(1,14)_=6.626, p=0.022, ŋ_p_^2^=0.321); TUG duration continued to improve in trials 17-20 compared to trials 5-8 of the session (F_(1,14)_=6.289, p=0.025, ŋ_p_^2^=0.310). Thus, overall, the gains in TUG duration across the three sessions, were on average 15.08% (range: 6%-30%) and 18.0% (range: 0%-37.3%) computed to before and after the transfer test segment, respectively."

• I would appreciate if the authors would also report normalized offline learning of the TUG duration (i.e. percent change from end of practice to beginning of retention test) between session 1, 2, and 3 (similar to the reporting of online gains).

**Response –** Given the reviewer’s comment we added the percent change from end of practice to beginning of retention test across the two intervals between sessions 1-3. In the methods section: "The offline, between sessions, gains in TUG duration were computed as percent change differences between the final trial of sessions 1 and 2 and the corresponding second trial of the next session, relative to performance in the first trial of session-1."

And we added, to the results section, the following statements: "The offline, between sessions, differences in TUG duration were -1.5 ± 3.8 percent for the interval between session-1 and session-2.” And “The offline differences in TUG duration from the end of session-2 to the beginning of session-3 were on average -4.0 ± 4.4 percent."

The corresponding percentages for the video data are now reported in the supplementary materials **(*Practice effects were retained across sessions (video)*)**:

"The offline, between sessions, differences in TUG duration were -1.3 ± 4.8, percent for the interval between session-1 and session-2." And "The offline differences in TUG duration from the end of session-2 to the beginning of session-3 were -1.6 ± 4.4, percent."

Figure:
• I agree with the authors that the trial by trial performance should be reported. However, I think that including additional bar charts showing (normalized) on- and offline learning may improve the results.

**Response –** Given the reviewer’s comment, we added a bar graph figure as additional supplementary figure to show the online and offline performance changes according to the videos and sensors throughout the first and last 4 trials segment of each session.

Fig S2: Performance changes within and between segments/sessions. A) According to the videos; B) according to the IMUs. */**/***- p<0.05, p<0.01, p<0.001(repeated measures ANOVA's.

• If the data of the transfer test are not reported in the paper I would suggest to remove them from figure 2.

**Response –** We think that it is necessary to represent the transfer test interval in figure 2 in order to maintain the correspondence to figure 1B (the overall plan).

Reviewer #2:

The authors comprehensively addressed all my concerns and I am happy to endorse the publication of this interesting and important article
**Response –** We thank the reviewer.
